# 

**Published:** 1883-06

**Authors:** 


### CONTENTS:

PAGE.

ORIGINAL COMMUNICATIONS:

Intra-Cranial Tumor. By William Pepper, M. D., LL. D., . . . . . . 481
Contagium Animatum. By Frederick Peterson, M. D., . . . . . . . 489

CLINICAL REPORTS:

Scirrhous Cancer of the Rectum in a Boy Eighteen Years of Age. By Herman Hayd, M. R. C. S., England, . . 497

FOREIGN CORRESPONDENCE, . . 499

SELECTIONS:

Treatment of Typhoid Fever by Antiseptics, Especially Carbolic and Salicylic Acids, 507
Syphilitic Re-infection of Husband and Wife, . . . . . . . . 510
Oleoresin of Male Fern: Increasing its Efficacy against Tape Worm, . . . 511
Recent Fracture of Skull with Depression, 512
Venereal and Common Warts, . . . 512
Ergot in Skin Diseases, . . . . 512
Hot Water in Therapeutics, . . . . 513
The Management of Strabismus Convergens. By B. St. John Roosa, M. D., LL. D., 516
The New Medical Code, . . . . . 517
Reduction of Dislocated Femur, . . . 518
White Lead Paint in Erysipelas, . . . 519
Surgical Expedients in Emergencies, . . 520

EDITORIAL:

Bacillus Tuberculosis, . . . . . . 522
Editorial Notes, . . . . . . . 523
Obituary, . . . . . . . . . 524

REVIEWS:

A Practical Treatise on Diseases of the Skin, for the use of Students and Practitioners, 525
The Dispensatory of the United States of America, . . . . . . . . 526
Aids to Medicine, Part No. 1. The General Diseases: Diseases of the Lungs, Heart, Blood Vessels and Liver, . . . 527
The Practitioner's Ready Reference Book, a Handy Guide in Office and Bed-side Practice, . . . . . . . . 527
Handbook of the Diagnosis of Treatment of Diseases of the Throat, Nose and Naso-pharynx, . . . . . . . . 528
Scrofula and its Gland Diseases, . . . 528

